# Will Memantine Exacerbate Seizures in People With Epilepsy? A Prospective Cohort Study

**DOI:** 10.1002/acn3.70262

**Published:** 2025-11-25

**Authors:** Peiyu Wang, Lu Lu, Hui Gao, Qi Zhang, Jing Xiao, Josemir W. Sander, Weixi Xiong, Dong Zhou

**Affiliations:** ^1^ Department of Neurology West China Hospital of Sichuan University Chengdu China; ^2^ Queen Square Institute of Neurology, Queen Square London WC1N 3BG, & Chalfont Centre for Epilepsy Chalfont St Peter UK

**Keywords:** anti‐dementia treatment, cognition, comorbidity, dementia, seizure

## Abstract

**Objective:**

To evaluate whether add‐on memantine would exacerbate seizures in people with epilepsy.

**Methods:**

This was a prospective cohort study. People with epilepsy diagnosed with cognitive impairment were consecutively invited. Those who agreed were followed up for at least 24 weeks. Participants were required to be adherent to their antiseizure medication regimens, which had remained unchanged for at least 24 weeks before recruitment. Participants were prescribed memantine and categorized according to their treatment decisions (memantine group vs. non‐memantine group). Primary outcome measures were the occurrence of new‐onset bilateral tonic–clonic seizure, new‐onset status epilepticus, and increased seizure frequency (≥ 25%). The incidence of other adverse events (AEs) and 24‐week retention rate were secondary outcome measures.

**Results:**

Two hundred and eleven participants were enrolled (median age, 63; male, 65%). Among them, one hundred and one (48%) started memantine. Baseline seizure characteristics were comparable between the two groups. During follow‐up, no new‐onset bilateral tonic–clonic seizure was recorded; there was one status epilepticus in the non‐memantine group. Increased seizure frequency was reported by 12 (6%) participants (memantine vs. non‐memantine, seven vs. five). Other AEs in the memantine group included headache (7%), dizziness (5%), fatigue (4%), drowsiness (2%), and anorexia (2%). The 24‐week retention rate was 91%.

**Interpretation:**

Adjunctive memantine was well tolerated in people with epilepsy regarding seizure control and other AEs. Previous warnings might have been disproportionate. Large‐scale, randomized trials are required to confirm this and further elucidate its efficacy profiles in epilepsy.

**Abbreviations**
AChI: acetylcholinesterase inhibitors
ADL: activities of daily living
AE: adverse event
ASM: antiseizure medication
BTCS: bilateral tonic–clonic seizure
EEG: electroencephalography
IQR: interquartile range
MMSE: mini‐mental status examination
MoCA: Montreal cognitive assessment
MRI: magnetic resonance imaging
NMDA: N‐Methyl D‐Aspartate
RCT: randomized controlled trial
STROBE: strengthening the reporting of observational studies in epidemiology

## 1 Introduction

Cognitive disorders are increasingly prevalent in people with epilepsy as the global population ages [1, 2, 3]. Accumulating studies have shown that people with epilepsy are at an enhanced risk of developing mild cognitive impairment or dementia [4, 5, 6, 7]. Seizures and subclinical epileptiform activities were found to be associated with the severity and progression of cognitive impairment in Alzheimer's disease [8]. The bidirectional link between these two conditions requires a more comprehensive management paradigm that emphasizes both seizure control and cognitive outcomes.

In practice, electing an appropriate cognitive enhancer could be challenging for people with an existing susceptibility to unprovoked seizures [9]. Memantine and acetylcholinesterase inhibitors (AChIs), such as donepezil, are the most widely used anti‐dementia agents [10]. Both types carry a warning for use in people with epilepsy due to their potential association with a lowered seizure threshold, for which memantine seems to be safer in people with epilepsy [11, 12, 13, 14]. The conflict between potential cognitive benefits and seizure risks could raise concerns. For instance, delaying the initiation of memantine in people with epilepsy due to concerns of seizure control. The warnings on memantine were primarily based on evidence from preclinical studies and individual case reports [15, 16]. The randomized‐controlled trials (RCTs) of memantine did not involve people with seizure disorders [9, 11, 12]. Several studies have supported the safety of memantine for use in people with epilepsy; however, the evidence is preliminary and limited by the small sample size and inadequate study design (e.g., unmatched baseline seizure‐control profiles between groups) [17, 18].

Due to the specific cautions, memantine has not been assessed to a larger extent nor fully extrapolated to clinical practice in the epilepsy population. Therefore, this study was conducted to provide evidence on the effect of memantine on seizure control, along with its overall safety in people with epilepsy.

## 2 Methods

### 2.1 Study Design and Ethical Approval

This study was a prospective, single‐center, observational assessment of the safety of memantine in individuals with epilepsy who were taking antiseizure medications (ASMs)—the setting was West China Hospital Neurology Centre. This study was performed in line with the principles of the World Medical Association Declaration of Helsinki. The study was approved by the Ethical Committee of West China Hospital (2024[1834]). The protocol was registered and is accessible on the Chinese Clinical Registry website (chictr.org.cn, ChiCTR2400092752). Participants provided written informed consent. We followed the Strengthening the Reporting of Observational Studies in Epidemiology (STROBE) reporting guidelines [19].

### 2.2 Participants

People who met the inclusion criteria and agreed to participate were consecutively enrolled from September 2023 to December 2024.

The inclusion criteria were:

1. diagnosed with epilepsy according to the 2014 International League Against Epilepsy (ILAE) definition [20];
2. diagnosed with cognitive impairment, defined as cognitive decline that interferes with activities of daily living (ADL). Cognitive impairment was determined by: (a) scoring ≤ 26 in the Mini‐mental status examination (MMSE) or (b) scoring ≤ the education‐adjusted norm in the Montreal Cognitive Assessment (MoCA), and activities of daily living were assessed using the ADL scale [21], according to the published Chinese guidelines [22, 23, 24].
3. aged ≥ 18 years;
4. had been continuously prescribed ASMs for at least one year and were on stable ASM treatment with unchanged dosage and types for at least the previous 24 weeks.

Exclusions were: (1) history of ischemic or hemorrhagic stroke, traumatic injury with subsequent loss of consciousness, brain surgery, cerebral infection, brain tumor, psychosis, or other major systemic illnesses within the previous six months; (2) history of substance abuse within the previous two years; (3) history of use of memantine or other N‐Methyl D‐Aspartate (NMDA) receptor antagonists; (4) irregular adherence to treatment or follow‐up. Those on concurrent AChIs were not excluded if they were on stable doses during the previous 24 weeks.

Adjunctive memantine was recommended to eligible participants at the baseline visit. Potential benefits and risks were discussed, and people were enrolled after providing informed consent. Those who chose to start with add‐on memantine were categorized as the exposure group (memantine group), while the remaining participants were classified as the control group (non‐memantine group).

### 2.3 Follow‐Up Procedure

#### 2.3.1 Baseline

The participants were evaluated clinically by a neurologist specializing in epilepsy (D. Z. & L. L.). They underwent a brain MRI scan, a 30‐min interictal scalp EEG, and a neuropsychometric assessment. This assessment included MMSE and MoCA for global cognitive performance, a test battery for individual domains of cognition, the generalized anxiety disorder 7‐item scale and neurological disorders depression inventory in epilepsy for screening anxiety and depression symptoms. A trained neuropsychologist performed the neuropsychometric assessments (P. W.).

#### 2.3.2 Memantine Titration

Memantine hydrochloride tablets were started at 5 mg once daily, slowly titrated to 10 mg once daily during the first week, and maintained at 10 mg once daily until discontinuation.

#### 2.3.3 Follow‐Up

Participants were followed for 24 weeks, with visits at four‐week intervals. Following the 24‐week follow‐up period, participants on memantine were followed up every 12 weeks until the study was discontinued. Paper seizure diaries were used to record seizure type and the corresponding frequency. Participants were instructed to complete the diary from the baseline visit to discontinuation or the most recent follow‐up. The caregiver (s) would be instructed to complete the diary if the participant was considered incapable of providing reliable records at baseline. At each follow‐up, seizure frequency and seizure type were recorded according to the diary from each participant. Participants and their caregiver (s) were interviewed about any other adverse events (AEs).

No changes were made to the ASM prescriptions during the study period. Adjustments to ASM regimens were allowed only when necessary for the evaluation of seizure control.

The dosage of memantine would be reduced to 5 mg once daily if any moderate or severe AE occurs after the initiation of memantine or if the seizure frequency increases and is considered relevant to memantine. Memantine was withdrawn if (1) moderate AEs continue despite dose reduction to 5 mg daily; (2) new‐onset BTCS or status epilepticus occurs; (3) other moderate or serious AEs occur and entail immediate discontinuation; or (4) the participant deems the treatment unsatisfactory.

### 2.4 Outcome

The primary outcomes were changes in seizure severity or frequency. Seizure exacerbation was defined as new‐onset episodes of bilateral tonic–clonic seizure (BTCS) or status epilepticus. Baseline seizure frequency was determined by calculating the average weekly seizure count over the 24‐week period preceding enrollment. Follow‐up seizure frequency was determined analogously over the 24‐week observation period after enrollment. Participants were considered to have an increased seizure frequency if the follow‐up seizure frequency surpassed the baseline seizure frequency by more than 25%. The proportions of participants experiencing seizure exacerbation and increased seizure frequency were compared between the two groups.

The secondary outcomes were the change in seizure frequency after memantine add‐on (i.e., the discrepancy between follow‐up seizure frequency and baseline seizure frequency in participants receiving add‐on memantine), the incidence of other AEs, and the 24‐week retention rate, defined as the proportion of participants who completed the 24‐week treatment period.

### 2.5 Statistical Analysis

Categorical variables were shown in proportions, and continuous variables were shown in the median (interquartile range [IQR]) or mean (SD). We used the Chi‐squared test, Fisher exact test, Mann–Whitney U test, Wilcoxon signed‐rank test, Kruskal‐Wallis test, analysis of variance (ANOVA), and independent t‐test to compare variables between groups when appropriate. Statistical tests were 2‐tailed, and α = 0.05 was used to determine significance. Chi‐squared tests, Fisher exact tests, Mann–Whitney U tests, Kruskal‐Wallis tests, analysis of variance (ANOVA), and independent t‐tests were performed using SPSS statistics software (version 26.0) and R software (version 4.5.1).

## 3 Results

Two hundred and eleven people were recruited (Table 1). Around two‐thirds of the participants were male, and the median age was 63 years. One hundred and one (48%) started adjunctive memantine (memantine group) after a recommendation, and the others had chosen not to. Age, sex, and years of education were comparable between groups. Participants who initiated memantine were more likely to present subjective cognitive complaints (*p* < 0.001), depression, and/or anxiety symptoms than their counterparts (*p* = 0.007). MMSE (*p* < 0.001) and MoCA (*p* = 0.001) scores showed greater impairment in global cognitive performance among participants treated with memantine than among those without. Reasons for refusal to start memantine included concerns about increased risks of seizures (*n* = 60), self‐perceived intact cognitive performance (*n* = 24), concerns about other potential drug AEs (*n* = 21), and concerns related to financial issues and personal beliefs (*n* = 5).

**TABLE 1:** Baseline Characteristics.

	Memantine (*n* = 101)	Non‐memantine (*n* = 110)	*P* ^a^
**Demographics**			
Age, median years (IQR)	64 (13)	62 (12)	
Male/Female, n	64/37	73/37	
Education, median years (IQR)	9 (6)	9 (6)	
**Comorbidity**			
Hypertension, *n* (%)	38 (38)	32 (29)	
Diabetes mellitus, *n* (%)	23 (23)	25 (23)	
Insomnia, *n* (%)	42 (42)	34 (31)	
MMSE, median scores (IQR)	23 (5)	25 (4)	< 0.001*
MoCA, median scores (IQR)	18 (6)	20 (5)	0.001*
Subjective Cognitive Complaint, *n* (%)	95 (94)	67 (61)	< 0.001*
Depression/Anxiety symptom, *n* (%)	30 (30)	15 (14)	0.007*
**Medical History**			
Cerebrovascular event, *n* (%)	21 (21)	12 (11)	0.058
Head trauma, *n* (%)	10 (10)	21 (19)	0.079
Cerebral surgery, *n* (%)	5 (5)	13 (12)	0.088
Cerebral infection, *n* (%)	6 (6)	7 (6)	
Brain tumor, *n* (%)	0	4 (4)	

^a^ Only *P*< 0.1 were shown. Significant *P*< 0.05 were shown with asterisks and bolded.

Baseline seizure characteristics are presented in Table 2. Three‐quarters of the participants had at least one seizure during the previous 24 weeks before the baseline period. Those on memantine had a significantly later onset of their first seizures than those in the non‐memantine group (*p* = 0.003). Participants without memantine had a longer course of epilepsy than participants in the memantine group (*p* = 0.015). Baseline seizure frequency, seizure type, and history of previous BTCS and status epilepticus were comparable between groups.

**TABLE 2:** Epilepsy characteristics.

	Memantine (*n* = 101)	Non‐memantine (*n* = 110)	*P* ^a^
Age of onset, median years (IQR)	60 (15)	56 (13)	0.003*
Epilepsy duration, median months (IQR)	30 (58)	48 (98)	0.015*
**Seizure type**			
Focal, *n* (%)	80 (79)	84 (76)	
Generalized, *n* (%)	2 (2)	1 (1)	
Focal and generalized, *n* (%)	2 (2)	1 (1)	
Unknown, *n* (%)	17 (17)	24 (22)	
**Etiology**			
Structural, *n* (%)	36 (36)	46 (42)	
Infectious, *n* (%)	2 (2)	5 (4)	
Unknown, *n* (%)	63 (62)	59 (54)	
**Seizure Frequency**			
Baseline weekly seizure frequency, median times (IQR)	0.083 (0.333)	0.083 (0.417)	
Baseline seizure frequency, median times/24 weeks (IQR)	2 (8)	2 (10)	
0, *n* (%)	28 (28)	27 (25)	
1 ~ 6, *n* (%)	45 (45)	47 (43)	
7 ~ 24, *n* (%)	19 (19)	22 (20)	
25 ~ 144, *n* (%)	8 (8)	12 (11)	
> 144, *n* (%)	1 (1)	2 (2)	

^a^ Only *P*< 0.1 were shown. Significant *P*(< 0.05) were shown with asterisks and bolded.

**FIGURE 1:**
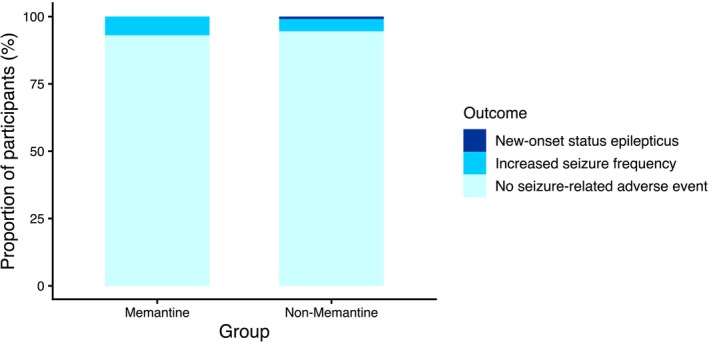
**Seizure‐related adverse events**. Proportions of participants reporting increased seizure frequency, new‐onset BTCS, and new‐onset status epilepticus were comparable between the two groups. No event of new‐onset BTCS was recorded.

By the end of the 24‐week follow‐up, one participant reported exacerbated seizures, and 12 (6%) participants reported increased seizure frequency (Figure 1). No significant difference was seen in the occurrence of increased or exacerbated seizures between the groups (Table 3).

**TABLE 3:** Incidence of increased seizure frequency, new‐onset BTCS, new‐onset status epilepticus, and other AEs.

	Memantine (*n* = 101)	Non‐memantine (*n* = 110)
# of participants reporting any AE, *n* (%)	24 (24)	17 (15)
Increased seizure frequency, *n* (%)	7 (7)	5 (5)
New‐onset BTCS, *n* (%)	0	0
New‐onset status epilepticus, *n* (%)	0	1 (1)
Serious AEs, *n* (%)	2 (2)	2 (2)
Other mild or moderate AEs, *n* (%)	19 (19)	12 (11)
Headache, *n* (%)	7 (7)	4 (4)
Dizziness, *n* (%)	4 (5)	1 (1)
Fatigue, *n* (%)	4 (4)	4 (4)
Drowsiness, *n* (%)	2 (2)	1 (1)
Anorexia, *n* (%)	2 (2)	1 (1)
Constipation, *n* (%)	0	1 (1)

In the memantine group, no significant difference (*p* = 0.275) was observed between baseline (median, 0.083) and follow‐up weekly seizure frequencies (median, 0.083). Seven participants (7%) experienced increased seizure frequency (participants A, B, C, D, E, F, and G): participant A, B, C, and D had breakthrough seizures due to incidental ASM nonadherence; participant E had had drug‐resistant epilepsy for 1 year and was having weekly focal autonomic seizures with focal‐to‐bilateral tonic–clonic seizures at the time of recruitment, and the weekly seizure frequency increased by a third after initiation of adjunctive memantine (0.833 at baseline vs. 1.083 at the end of follow‐up); participant F, having had focal‐to‐bilateral tonic–clonic seizures for 10 years and achieved stable seizure control with one seizure per 3–4 months, had a 50% increase in weekly seizure frequency at the end of follow‐up (0.083 at baseline vs. 0.125 at the end of follow‐up); participant G, having had focal nonmotor seizures for 18 months and remained on levetiracetam monotherapy for 12 months before recruitment, had a 25% increase in weekly seizure frequency (0.167 at baseline vs. 0.208 at the end of follow‐up). These participants remained on their original ASM regimen and continued memantine at 10 mg once daily. No new‐onset BTCS or status epilepticus was recorded.

We recorded four serious AEs. Of the participants on memantine, one had bacterial pneumonia, and another had a new diagnosis of colorectal carcinoma. These serious AEs were considered not relevant to memantine. In controls, one had new‐onset nonconvulsive status epilepticus and the other viral pneumonia. Other than seizure‐related AEs, 23 (11%) participants reported mild or moderate AEs at the end of the 24‐week follow‐up. These AEs led to a dose reduction in five participants on memantine, who reported complete headache resolution and continued treatment. Four participants in the memantine group discontinued memantine as they continued to experience intolerable headache, dizziness, and anorexia despite dose reduction. No significant difference was observed in the proportion of participants reporting any AEs between groups (*p* = 0.163) (Table 3).

**FIGURE 2:**
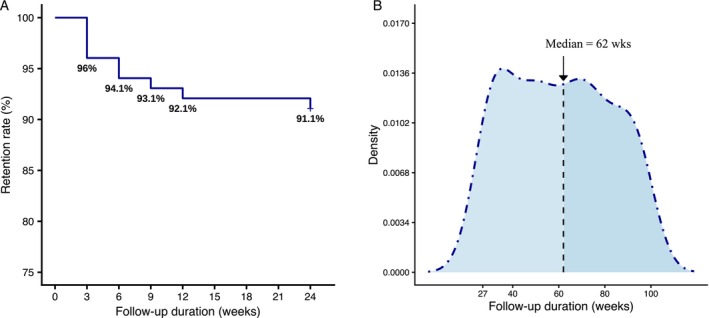
Retention rate and follow‐up duration. (A) The 24‐week retention rate of memantine; (B) Distribution of follow‐up duration of the participants taking memantine. The median duration was 62 weeks.

The 24‐week retention rate on memantine was 91%. Nine participants had discontinued memantine by the end of the 24‐week follow‐up. Reasons for withdrawal were intolerable AEs (*n* = 4) and failing personal treatment expectations (*n* = 5). Following the 24‐week follow‐up, all remaining participants (*n* = 92) continued memantine until the latest follow‐up in July 2025. The treatment duration ranged from 27 to 95 weeks (median, 62 weeks) (Figure 2A,B).

During the study period, 146 (69%) participants were on monotherapy for seizure control, with levetiracetam being the most common ASM (85%). ASM prescriptions adhered to the current standard and remained unchanged throughout the study course despite reported changes in seizure severity and frequency. Participants from the memantine group were more frequently prescribed antidepressants than those from the non‐memantine group (12% vs. 1%, *p* = 0.001). Concurrent use of sleep aids, AChIs, and medications for hypertension and type 2 diabetes mellitus was comparable between groups (Table 4).

**TABLE 4:** Medication treatment, EEG, and MRI profiles.

	Memantine (*n* = 85)	Non‐memantine (*n* = 93)	*P* ^a^
**ASM**			
Monotherapy, *n* (%)	75 (74)	71 (65)	
Levetiracetam, *n* (%)	65 (87)	59 (83)	
Lacosamide, *n* (%)	5 (7)	6 (8)	
Valproate, *n* (%)	3 (4)	1 (1)	
Oxcarbazepine, *n* (%)	2 (3)	5 (7)	
Polytherapy, *n* (%)	26 (26)	39 (35)	
Drug‐resistant epilepsy, *n* (%)	14 (14)	16 (15)	
**Co‐administered Neuroactive Drugs**			
Sleep‐aid, *n* (%)	10 (10)	6 (5)	
Anti‐depressant, *n* (%)	12 (12)	1 (1)	0.001*
AChI, *n* (%)	4 (4)	4 (4)	
**EEG**			
Abnormal, *n* (%)	62 (61)	52 (47)	**0.053**
Slowing, *n* (%)	50 (49)	45 (41)	
IED, *n* (%)	30 (30)	27 (25)	
**MRI**			
Lesional, *n* (%)	43 (43)	54 (49)	

^a^ Only *P*< 0.1 were shown. Significant *P*< 0.05 were shown with asterisks and bolded.

## 4 Discussion

This study showed that the use of memantine in people with epilepsy did not significantly raise the risk of seizure exacerbation or increase seizure frequency as an add‐on treatment. It was generally well tolerated, with a high retention rate.

Memantine use in people with epilepsy has been cautioned based on inconclusive evidence. Previous clinical reports indicated that incident seizures in a small number of people taking memantine accounted for a prominent portion of the reported AEs [14, 25]. However, this effect was not significant compared with placebo in RCTs [26]. Interpreting seizures in people taking memantine is challenging, as cognitive disorders are linked to an increased risk of seizures. Two RCTs involving a small number of adults with epilepsy supported a favorable safety profile for memantine when administered 10 mg once daily and 10 mg twice daily [17, 18]. In our study, seizure‐related AEs were rare and were not associated with memantine use, as the incidence of increased seizure frequency was comparable between participants with and without memantine. The recorded AEs of increased seizure frequency were considered incidental or attributable to missed ASM doses. The only event of new‐onset status epilepticus occurred in a participant without adjunctive memantine. These findings indicate that add‐on memantine is unlikely to impact seizure control negatively. Given the small sample size, more large‐scale studies are needed to confirm these results.

Concerns about seizure exacerbation have affected further investigations into the safety and efficacy of memantine in people with epilepsy. Its potential proconvulsive and anticonvulsive effects have been observed in various seizure models and in cerebral tissue cultures. Epilepsy is a heterogeneous condition, and memantine might affect different epilepsy types and etiologies differently. For instance, one RCT suggested memantine was safe and effective in children with developmental and epileptic encephalopathy [27]. Only a few trials have evaluated the efficacy of memantine in treating cognitive impairment in epilepsy, in which memantine was found to improve cognitive performance and memory [17, 18, 28]. In the era of precision medicine, memantine might be considered a candidate for improving neuropsychiatric outcomes in epilepsy. Our study provided safety evidence in support of a larger‐scale assessment of memantine in epilepsy.

The potential for increased seizure risk from cognitive‐enhancing drugs often causes undue concern, and current guidelines offer little guidance [29, 30, 31]. While optimizing ASM strategies can improve outcomes [3, 32, 33], this approach has limitations, especially in older people, as cognitive impairment can result from several pathophysiological processes beyond just seizure burden and ASM effects [12, 34]. Memantine, a validated treatment for several dementing disorders, presents a potential option. Our study shows that over half of the participants declined memantine, primarily due to concerns about increased seizure risk. This likely extends to physicians, creating a barrier to its wider consideration.

Other AEs, such as headache, dizziness, and drowsiness, were consistent with previous RCTs in elderly populations [35]. All AEs were dose‐dependent and resolved at a lower dose or after discontinuation. One study reported that the most frequent AE in adults with epilepsy on memantine was dizziness, followed by somnolence and headache [17].

In clinical settings, polytherapy is expected among older adults. Our study in a geriatric population demonstrated that low‐dose memantine is compatible with ASMs, with types and doses aligning with current clinical practices. Levetiracetam monotherapy was the most common ASM choice in our cohort. We observed no significant drug–drug interactions. When combined with other neuroactive agents, memantine showed promising results in our cohort. Antidepressants were more frequently prescribed to participants starting adjunctive memantine, which was associated with incident seizures in previous studies [14, 36]. Despite this, the memantine group reported seizure outcomes similar to those of the non‐memantine group. Co‐administration with other neuroactive agents, such as sleep aids and acetylcholinesterase inhibitors (AChIs), was also well tolerated in our cohort.

Participants accepting memantine showed worse subjective and objective cognitive performance and complained more often about depression and/or anxiety symptoms, which could partly explain their compliance with the recommendation of add‐on memantine. This finding also implies that mental health screening should be incorporated into the routine management of elderly epilepsy.

Our study has limitations. Firstly, heterogeneity in ASM treatment among participants persisted even after excluding participants with individualized ASM adjustments to improve uniformity, with variations in ASM types and dosages remaining. These differences could impact our results despite comparable ASM prescriptions between groups. This exclusion limits the generalizability of our findings. Secondly, given the observational design and limited sample size in an elderly Chinese population, caution is warranted when extrapolating these findings to broader populations. Thirdly, due to the limited sample size, the incidence of seizure‐related and other severe AEs may have been underestimated. We couldn't assess the risk of seizure‐related AEs based on seizure type, etiology, or severity. Additionally, our safety measures relied on self‐reported events or changes in seizure frequency, which might be unreliable. Lastly, the study was relatively short. However, as it was primarily focused on the safety of memantine regarding its impact on seizure frequency and severity, such findings should have been evident early in the study, making the duration less relevant.

Despite these limitations, our results offer new insights into the safety of memantine add‐on therapy in epilepsy. However, the safety and efficacy of memantine require further demonstration through RCTs in people with epilepsy and cognitive impairment, as well as in people with dementia experiencing incident seizures.

Our findings suggest that low‐dose memantine is generally safe and well tolerated in older adults with epilepsy when combined with ASMs. Concerns about the use of memantine in seizure disorders may have been disproportionate to the actual risks. More RCTs are required to clarify the safety and efficacy profiles of adjunctive memantine in epilepsy.

## Author Contributions

D.Z. and L.L. conceptualized the study design. P.W. and L.L. performed the search, data interpretation, and drafted and revised the manuscript. P.W., Q.Z., and J.X. performed participant recruitment and follow‐up. D.Z. performed clinical evaluation. P.W., Q.Z., J.X., and L.L. collected clinical and neuropsychological data. P.W. performed data analysis. H.G. and J.W.S. were involved with data interpretation and reviewed the manuscript. W.X. and D.Z. supervised the study.

## Funding

This work was supported by West China Hospital, Sichuan University (2024HXBH095, ZYGD23032). National Key Research and Development Program of China (2021YFC2401204). The National Natural Science Foundation of China (U21A20393).

## Conflicts of Interest

The authors declare no conflicts of interest.

## Data Availability

The data that support the findings of this study are available on request from the corresponding author. The data are not publicly available due to privacy or ethical restrictions.
